# Maternal embryonic leucine zipper kinase is a novel target for diffuse large B cell lymphoma and mantle cell lymphoma

**DOI:** 10.1038/s41408-019-0249-x

**Published:** 2019-11-18

**Authors:** Anke Maes, Ken Maes, Philip Vlummens, Hendrik De Raeve, Julie Devin, Vanessa Szablewski, Kim De Veirman, Eline Menu, Jerome Moreaux, Karin Vanderkerken, Elke De Bruyne

**Affiliations:** 1 Department of Hematology and Immunology, Myeloma Center Brussels, Vrije Universiteit Brussel, Brussels, Belgium; 2 Hematology, Department of Internal Medicine, Ghent University Hospital, Ghent, Belgium; 3 Department of Pathology, UZ Brussel, Vrije Universiteit Brussel, Brussels, Belgium; 4 Laboratory for Monitoring Innovative Therapies, Institute of Human Genetics, CNRS, Montpellier, France; 5 Department of Biopathology, CHU Montpellier, France

**Keywords:** Translational research, B-cell lymphoma

## Abstract

Diffuse large B cell lymphoma (DLBCL) and mantle cell lymphoma (MCL) are among the most aggressive B cell non-Hodgkin lymphomas. Maternal embryonic leucine zipper kinase (MELK) plays a role in cancer cell cycle progression and is associated with poor prognosis in several cancer cell types. In this study, the role of MELK in DLBCL and MCL and the therapeutic potential of MELK targeting is evaluated. MELK is highly expressed in DLBCL and MCL patient samples, correlating with a worse clinical outcome in DLBCL. Targeting MELK, using the small molecule OTSSP167, impaired cell growth and survival and induced caspase-mediated apoptosis in the lymphoma cells. Western blot analysis revealed that MELK targeting decreased the phosphorylation of FOXM1 and the protein levels of EZH2 and several mitotic regulators, such as Cdc25B, cyclin B1, Plk-1, and Aurora kinases. In addition, OTSSP167 also sensitized the lymphoma cells to the clinically relevant Bcl-2 inhibitor venetoclax by strongly reducing Mcl1 levels. Finally, OTSSP167 treatment of A20-inoculated mice resulted in a significant prolonged survival. In conclusion, targeting MELK with OTSSP167 induced strong anti-lymphoma activity both in vitro and in vivo. These findings suggest that MELK could be a potential new target in these aggressive B cell malignancies.

## Introduction

Non-Hodgkin lymphoma (NHL) is the most common hematological cancer, with diffuse large B cell lymphoma (DLBCL) and mantle cell lymphoma (MCL) among the most aggressive subtypes. Within the DLBCL subtype, different molecular subgroups are distinguished based upon the cell of origin, including Activated B cell (ABC)- and Germinal Center B cell (GCB)-DLBCL^1,2^. ABC-DLBCL has a worse clinical outcome compared with GCB-DLBCL^2^. Recently, a new distinct entity with poor prognosis was described as “the double/triple hit lymphomas”, with rearrangements of MYC and BCL-2 and/or BCL-6. In addition, patients with high expression of both Myc and Bcl-2, but lacking the identifiable rearrangements (called “double expressers”) also have a poor prognosis^3^. The standard-of-care treatment in these aggressive NHLs consists of the R-CHOP regimen (rituximab, cyclophosphamide, doxorubicin, vincristine, and prednisone). However, 30% of DLBCL patients and almost all MCL patients still relapse^1,4^. Therefore, investigation and development of new strategies are still required.

Maternal embryonic leucine zipper kinase (MELK), also known as pEg3 or murine protein K38 (MPK38), is a serine/threonine protein kinase that plays a role in several biological processes, such as stem cell phenotypes, mitotic progression, apoptosis, and tumorigenesis^5–7^. It has been suggested that MELK regulates the G2/M transition, by phosphorylating proteins that regulate G2/M cell cycle progression^8^. MELK is able to phosphorylate and activate the transcription factor FOXM1, which drives expression of several mitotic regulatory proteins, including Aurora kinases, cyclin B1, and Survivin^8,9^. Cdc25B, which is an activator of Cdk1 in early mitosis, can also be phosphorylated and activated by MELK^10–12^. In addition, enhancer of zeste homolog 2 (EZH2), c-Jun, p53, apoptosis signal-regulating kinase 1 (ASK1), DEPDC1, and SOX2 are also targets of MELK^5,7,13^. Several studies observed high MELK levels in different types of cancer and MELK overexpression is often associated with a poor prognosis^6,7,14–18^. Moreover, knockdown studies resulted in decreased survival of cancer cells^19,20^. The implication of MELK in tumor growth makes it an attractive therapeutic target for cancer therapy^7^. OTSSP167 is a potent MELK inhibitor and was shown to impair cancer growth in leukemia, myeloma, small cell lung cancer, neuroblastoma, prostate cancer, and kidney cancer cells^15,18,21–24^. As such, OTSSP167 is currently tested in different clinical trials in patients with solid tumors and refractory and relapsed leukemia^25^. In this study, the clinical relevance of MELK and the therapeutic potential of the MELK inhibitor OTSSP167 was investigated in DLBCL and MCL.

## Materials and methods

### Analysis of MELK gene expression levels

The publicly available gene expression profiling (GEP) datasets with gene expression data and survival data of 183 GCB-DLBCL patients, 167 ABC-DLBCL patients and 64 unclassified DLBCL patients (GSE10846)^26,27^, gene expression data of 33 B cell samples, 23 ABC-DLBCL patients, 29 GCB-DLBCL patients, and 3 unclassified DLBDL patients (GSE56315)^28^, gene expression data of 7 indolent and 15 aggressive MCL patients (GSE16455)^29^, and gene expression data of 13 human DLBCL cell lines and 5 MCL cell lines (GSE36133)^30^ were used. To minimize batch effects from the different experiments, raw CEL files were obtained from the Gene Expression Omnibus (GEO) and GCRMA-normalization on pooled CEL files was performed in R using bioconductor packages oligo and affy^31,32^. The following probeset was used: 204825-at.

### Patient biopsies and staining

Patient samples were collected at the Department of Biopathology in Montpellier (CHU Montpellier, France). Three-μm-thick sections from tissue microarrays containing three representative 0.6-mm cores of routinely processed tissues from 27 DLBCL patients treated with R-CHOP were included^33^. The quality of each tissue core was evaluated based on its morphology, using hematoxylin and eosin staining, and the percentage of CD20+ tumor cells. Only tissue cores with more than 50% CD20+ tumor cells were retained for immunohistochemical analysis. All samples were stained with MELK antibody (Sigma-Aldrich, Bornem, Belgium) using the automated immunostainer Benchmark XT (Roche Ventana, Basel, Switzerland). In addition, a CD20 staining was performed on consecutive slides. As a positive control, tonsils with secondary follicles were used. Immunostaining results (i.e., percentage of positive cells) were evaluated by an expert pathologist. The percentage of tumor cells with a staining of any intensity was determined at a magnification ×400 on a Leica DM2000 microscope. The counting was performed in hot-spot areas by means of an ocular grid. Patients were divided into good and poor prognosis groups based on the validated revised International Prognostic Index (R-IPI) score of the DLBCL patients. The study protocol was approved by the ethics committee of Montpellier and the informed consent was obtained from all subjects (DC-2013-2027).

### Cell culture

The human GCB-DLBCL cell lines SU-DHL-6, OCI-Ly1, and OCI-Ly7 were maintained in IMDM medium (Life Technologies) supplemented with 10% fetal calf serum (FCS) (Biochrom AG, Berlin, Germany) and 2 mM glutamine (Life Technologies, Gent, Belgium). ABC-DLBCL (U2932 and RI-1) and MCL cell lines (Jeko-1, Mino and Rec-1) were maintained in RPMI-1640 medium (Lonza, Basel, Switzerland) supplemented with 10% FCS and 2 mM glutamine. The murine DLBCL cell line (A20) was maintained in supplemented RPMI-1640 medium with 0.05 mM β-mercapto-ethanol (Sigma-Aldrich). Cells were cultured at 37 °C in a humidified 5% CO_2_ atmosphere. All cell lines were obtained from ATCC and regularly tested for mycoplasma contamination and checked for authenticity by STR profiling.

### Reagents

The MELK inhibitor OTSSP167 was obtained from MedChem Express (Bio-Connect, Huissen, The Netherlands). Venetoclax and rituximab were obtained from Selleckchem (Bio-Connect) and doxorubicin hydrochlorate was purchased from Sigma-Aldrich. All agents were dissolved in DMSO for in vitro studies and OTSSP167 (hydrochloride) was dissolved in 5% glucose for the in vivo experiment.

### Quantitative real-time PCR

Total RNA was extracted using the Nucleospin RNA plus kit (Macherey-Nagel, Düren, Germany) and reverse transcription was performed using the Verso cDNA synthesis kit (ThermoFisher Scientific, Gent, Belgium), both according to manufacturer’s instructions. Quantitative real-time PCR was performed as previously described^34^. Primers for MELK were purchased from IDT (Leuven, Belgium) and primers for GAPDH were purchased from Qiagen (Venlo, The Netherlands). Primer sequences for MELK were as followed (5′–3′): forward: GTG CTA GAG ACA GCC AAC AA; reverse: CAG GCG ATC CTG GGA AAT TA.

### Western blot analysis

Cells were harvested, lysed and western blot was performed as previously described^34^. Antibodies were used against MELK (#2274), Aurora B kinase (#3094), cyclin B1 (#4138), pFOXM1 (#14170), EZH2 (#4905), Cdc25B (#9525), Plk-1 (#4535), Aurora A kinase (#3092), PARP (#9542), Mcl1 (#5453), pBcl-2 (#2827), Bcl-xL (#2764), caspase-3 (#9662) and β-actin (#4967) (all from Cell Signaling Technology, Leiden, the Netherlands) and FOXM1 (sc_376471), Bcl-2 (sc_492) and pBcl-xL (sc_101644) (Santa Cruz, Heidelberg, Germany). Quantification of the different protein levels was performed in Image J, according to manufacturer’s instructions.

### Cell viability assay

The CellTiter-Glo Luminiscent Viability assay (Promega, Leiden, The Netherlands) was used to measure cell viability according to manufacturer’s instructions. 100,000 cells/ml were treated with the indicated agent and cell viability was measured after 48 h.

### Apoptosis assay

100,000 cells/ml were treated with the indicated agent(s) and apoptosis was measured after 48 h. Cells were harvested and apoptosis was quantified using an Annexin V/7’-AAD staining (BD Biosciences, Franklin Lakes, USA) and active caspase-3 staining (BD Biosciences), according to manufacturer’s instructions. Cells were analyzed by flow cytometric analysis (FACS Canto, BD Biosciences).

### BrdU cell proliferation assay

The BrdU Cell Proliferation Assay Kit (Cell Signaling Technology) was performed according to manufacturer’s instructions. 100,000 cells/ml were treated with the indicated agent for 24 h and the BrdU cell proliferation assay was performed after 3 h of BrdU labeling.

### Cell cycle analysis

100,000 cells/ml were treated with the indicated agent and cell cycle analysis was performed after 24 h. Propidium iodide (PI) staining was used to analyze cell cycle distribution. Cells were harvested and incubated for 10 min with a PI solution containing 1 mg/ml sodium nitrate (Merck KGaA, Darmstadt, Germany), 0.1% Triton-X (Merck), 100 µg/ml RNase A (Boehringer, Ingelheim, Germany), and 50 µg/ ml PI (Sigma-Aldrich). Cells were analyzed by flow cytometry.

### In vivo experiment

Female BALB/c mice were purchased from Charles River (Ecully, France). They were housed and maintained following the conditions approved by the Ethical Committee for Animal Experiments, Vrije Universiteit Brussel (license no. LA1230281, CEP no. 18-231-4). Mice were subcutaneously inoculated with 1 × 10^6^ A20 cells in the right flank as previously described^35^. When tumors were palpable, mice were treated three times a week via intravenous injection with OTSSP167 (10 mg/kg). Mice were sacrificed when the tumor reached a maximum volume of 1500 mm³. Tumor volume was calculated as followed: volume (mm³) = (length × width²)/2.

### Statistical analysis

Prognostic significance of MELK gene expression was calculated using the MaxStat R package. Statistical differences in overall survival was calculated by a log-rank test and survival curves were plotted using Kaplan–Meier method. Graphical and statistical analysis was performed using GraphPad Prism 5.01 software. Statistical significance (*p*-value of *p* < 0.05 was considered significant) was determined by a Mann–Whitney U-test (to compare two groups) and a one-way ANOVA with Bonferonni correction for multiple testing.

## Results

### MELK expression is upregulated in DLBCL and MCL, correlating with a poor survival in DLBCL

**Fig. 1:**
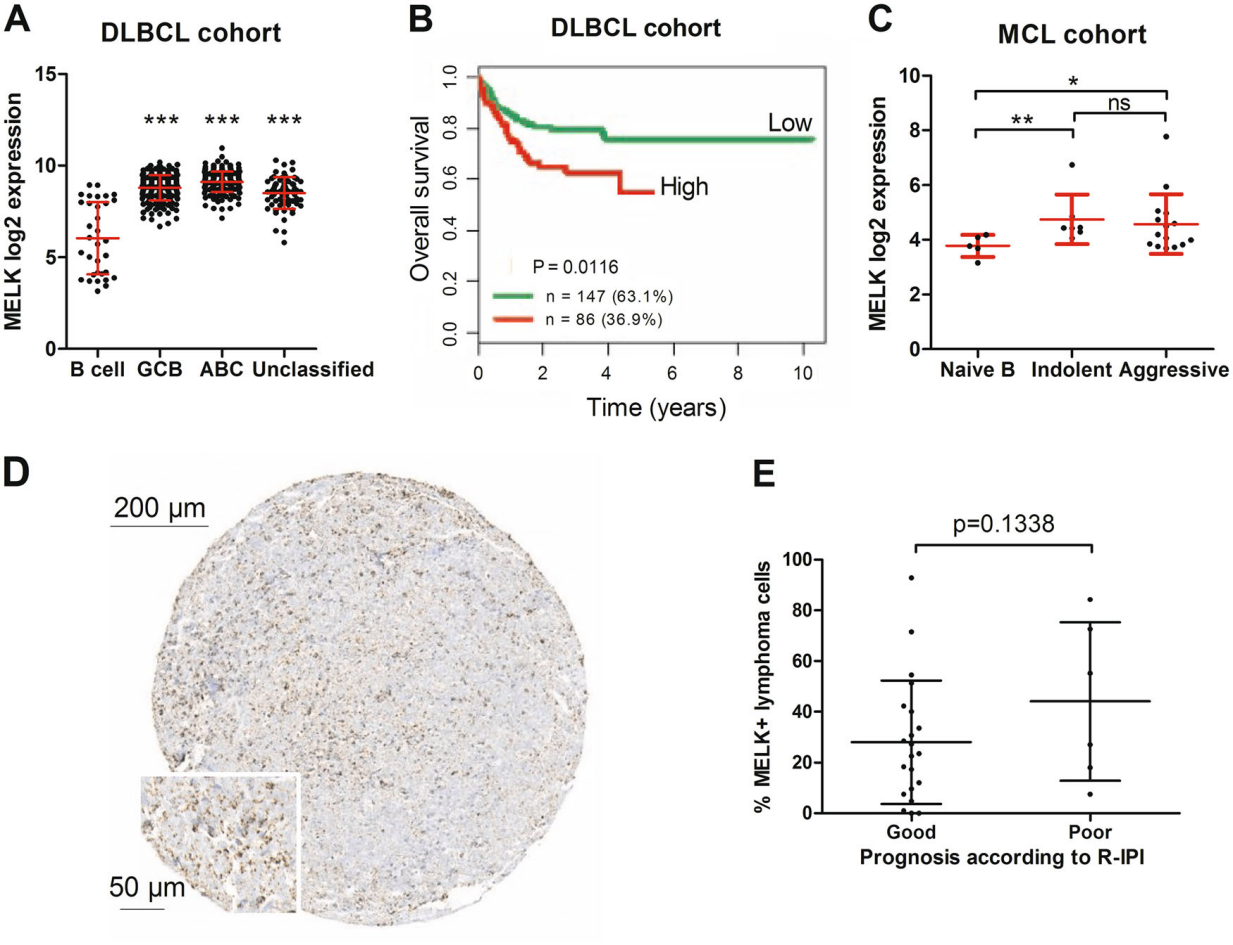
MELK mRNA expression is increased in DLBCL and MCL patients, which is associated with poor survival in DLBCL. **a** MELK gene expression levels of B cell samples (*n* = 33), patients with ABC-DLBCL (*n* = 190), patients with GCB-DLBCL (*n* = 212) and patients with unclassified DLBCL (*n* = 67) were obtained from the publicly available microarray datasets GSE10846 and GSE56315. Mean expression ± SD is shown in red. ***p < 0.001. **b** The prognostic value of MELK was determined in DLBCL patients (93 ABC, 108 GCB, and 32 unclassified DLBCL patients) receiving R-CHOP treatment from the Lenz cohort (*n* = 233) using Maxstat analysis (cut-off value used is 9.17). **c** MELK gene expression levels of patients with indolent (*n* = 7) and aggressive MCL (*n* = 15) were obtained from the GSE16455 dataset. Mean expression ± SD is shown in red. *p < 0.05 and **p < 0.01. **d** Immunohistochemical analysis of MELK expression in a DLBCL patient. A ×4 and ×20 magnification is shown. **e** Percentage of MELK positive lymphoma cells was counted in 27 DLBCL patients treated with R-CHOP and plotted against the estimated prognosis according to the R-IPI-score of the patients.

The potential clinical relevance of MELK in the aggressive lymphomas DLBCL and MCL was assessed using publicly available GEP datasets. MELK mRNA expression was examined using GEP data from B cell samples (*n* = 33), GCB-DLBCL (*n* = 212), ABC-DLBCL (*n* = 190), and unclassified DLBCL patients (*n* = 67). As shown in Fig. 1a, MELK expression was significantly increased in all DLBCL subtypes compared with normal B cell samples. Moreover, high MELK expression levels were associated with a significant worse survival in DLBCL patients (93 ABC-, 108 GCB-, and 32 unclassified DLBCL patients) receiving R-CHOP treatment (Fig. 1b). GEP data from naive B cells (*n* = 6), indolent MCL (*n* = 7), and aggressive MCL patients (*n* = 15) were used to investigate the MELK expression in MCL patients. Analysis of MELK mRNA expression in this MCL patient cohort revealed that both indolent and aggressive MCL primary samples have significant higher levels of MELK compared with the naive B cell samples (healthy counterparts of MCL cells^36^), with no significant difference in MELK mRNA expression between indolent and aggressive MCL patients (Fig. 1c).

Next, the prognostic value of MELK expression in DLBCL patients was examined. The protein expression of MELK was determined in a small patient cohort of DLBCL patient treated with R-CHOP (Fig. 1d). The percentage of MELK positive lymphoma cells was plotted against the patient’s prognosis according to their R-IPI. The trend of associating a poor prognosis and a higher amount of MELK positive cells is observed (Fig. 1e).

### Pharmacological inhibition of MELK using OTSSP167 induces apoptosis

To investigate MELK as a potential target in human DLBCL and MCL cell lines, MELK expression was first assessed in the GEP data of a large panel of human DLBCL and MCL cell lines. Significant higher MELK mRNA expression was observed in all DLBCL and MCL cell lines compared with the B cell samples (Supplemental Fig. 1A). These observations were also confirmed in a select panel of human DLBCL (both ABC- and GCB-subtype) and MCL cell lines, both at mRNA and protein levels (Supplemental Fig. 1B–C).

**Fig. 2:**
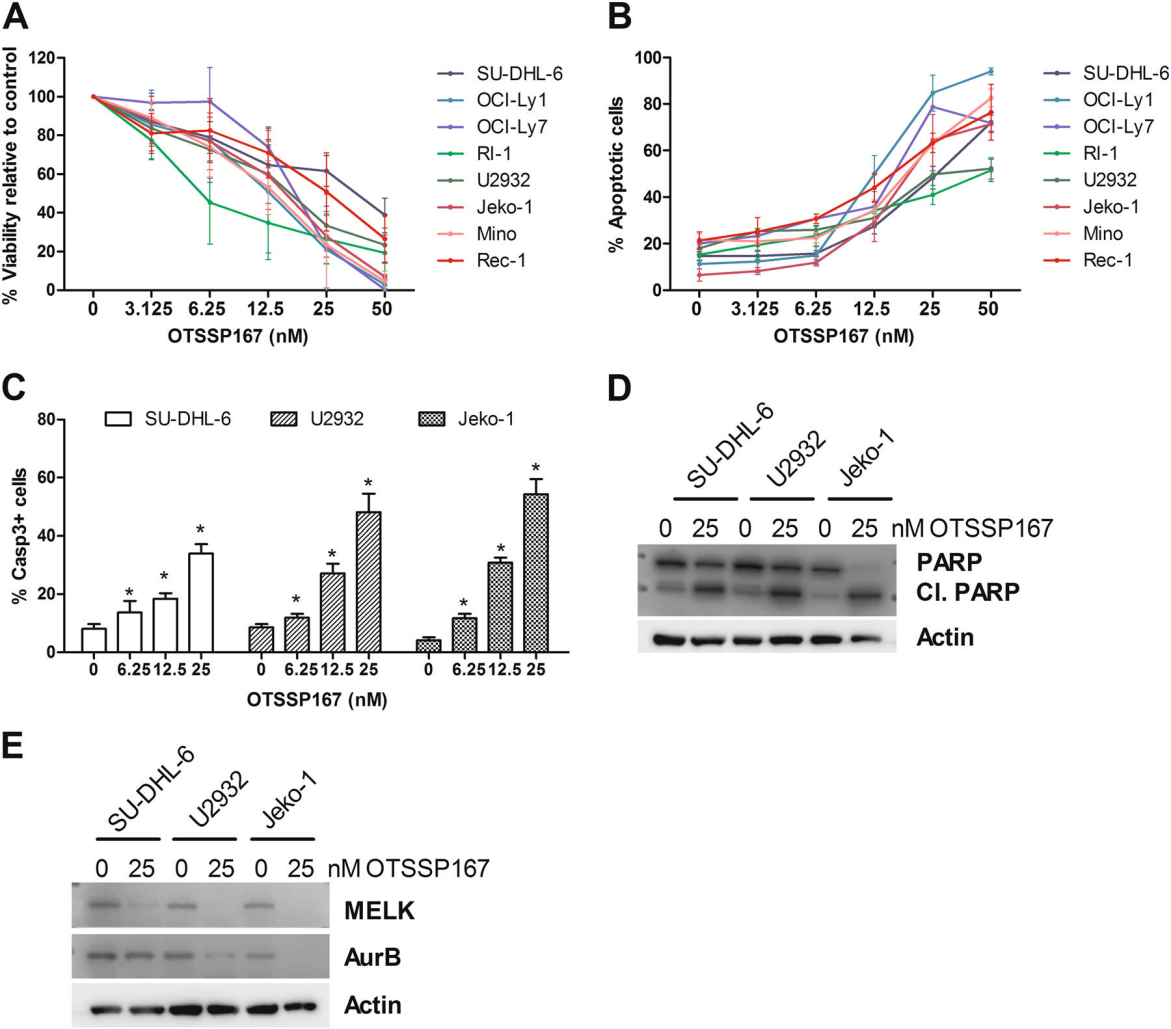
MELK inhibition, using OTSSP167 treatment, reduces lymphoma cell viability and induces caspase-mediated apoptosis. **a**–**c** DLBCL and MCL cell lines were treated for 48 h with OTSSP167 (3.125, 6.25, 12.5, 25, and 50 nM). The effect on viability was determined using a CellTiter-Glo assay (**a**). Results are shown as % viability relative to control. Results shown are mean ± SD of three independent experiments. The effect on apoptosis was determined using an Annexin V/7’-AAD staining followed by flow cytometric analysis (**b**). Percentage apoptotic cells are the sum of the percentage Annexin V and Annexin V/7’-AAD positive cells. Results shown are mean ± SD of three independent experiments. The percentage of caspase-3-positive cells was determined using active caspase-3 staining followed by flow cytometry analysis (**c**). Results shown are mean ± SD of three independent experiments. *p < 0.05 (**d**, **e**). Expression of PARP (**d**), MELK (**e**), and Aurora B kinase (AurB, **e**) was determined after 48 h of 25 nM OTSSP167 treatment using western blot. β-actin was used as loading control. One experiment representative of 3 is shown.

The effect of the pharmacological MELK inhibitor OTSSP167 was then investigated in the selected human DLBCL and MCL cell lines. First, the effect of the MELK inhibition on lymphoma cell viability was assessed. A dose-dependent decrease in viability was observed in all cell lines after 48 h of OTSSP167 treatment with an IC-50 value around 15 nM for most of the DLBCL cell lines, except for the RI-1 cells and SU-DHL-6 cells (Fig. 2a and Supplemental Table 1A). The ABC-DLBCL cell line RI-1 was found the most sensitive (IC-50 ≈ 6 nM), while the GCB-DLBCL cell line SU-DHL-6 was the least sensitive (IC-50 ≈ 30 nM). Among the MCL cell lines, the Mino and the Rec-1 cells were, respectively, the most sensitive (IC-50 ≈ 13 nM) and the least sensitive (IC-50 ≈ 25 nM). Importantly, no clear correlation between MELK protein expression and OTSSP167 sensitivity nor between the doubling time and OTSSP167 sensitivity was found (Supplemental Table 1, B–C). Then, the effect of MELK inhibition was determined on apoptosis by an Annexin V/7’-AAD and active caspase-3 staining. Consistent with the viability data, a dose-dependent increase in apoptotic cells was observed in all cell lines after 48 h of OTSSP167 treatment, which was associated with an increase in the amount of caspase-3-positive cells (Fig. 2b, c). In addition, western blot analysis demonstrated that OTSSP167 treatment resulted in a clear increase in cleaved poly ADP-ribose polymerase (PARP) protein levels after 48 h (Fig. 2d). Importantly, western blot analysis also confirmed the on-target effect of OTSSP167 as evidenced by clear reduction in MELK protein levels in the GCB-DLCBL cell line SU-DHL-6, the ABC-DLBCL cell line U2932 and the MCL cell line Jeko-1 and in Aurora B kinase protein levels in U2932 and Jeko-1 cells (Fig. 2e).

### OTSSP167 treatment affects lymphoma cell cycle progression

**Fig. 3:**
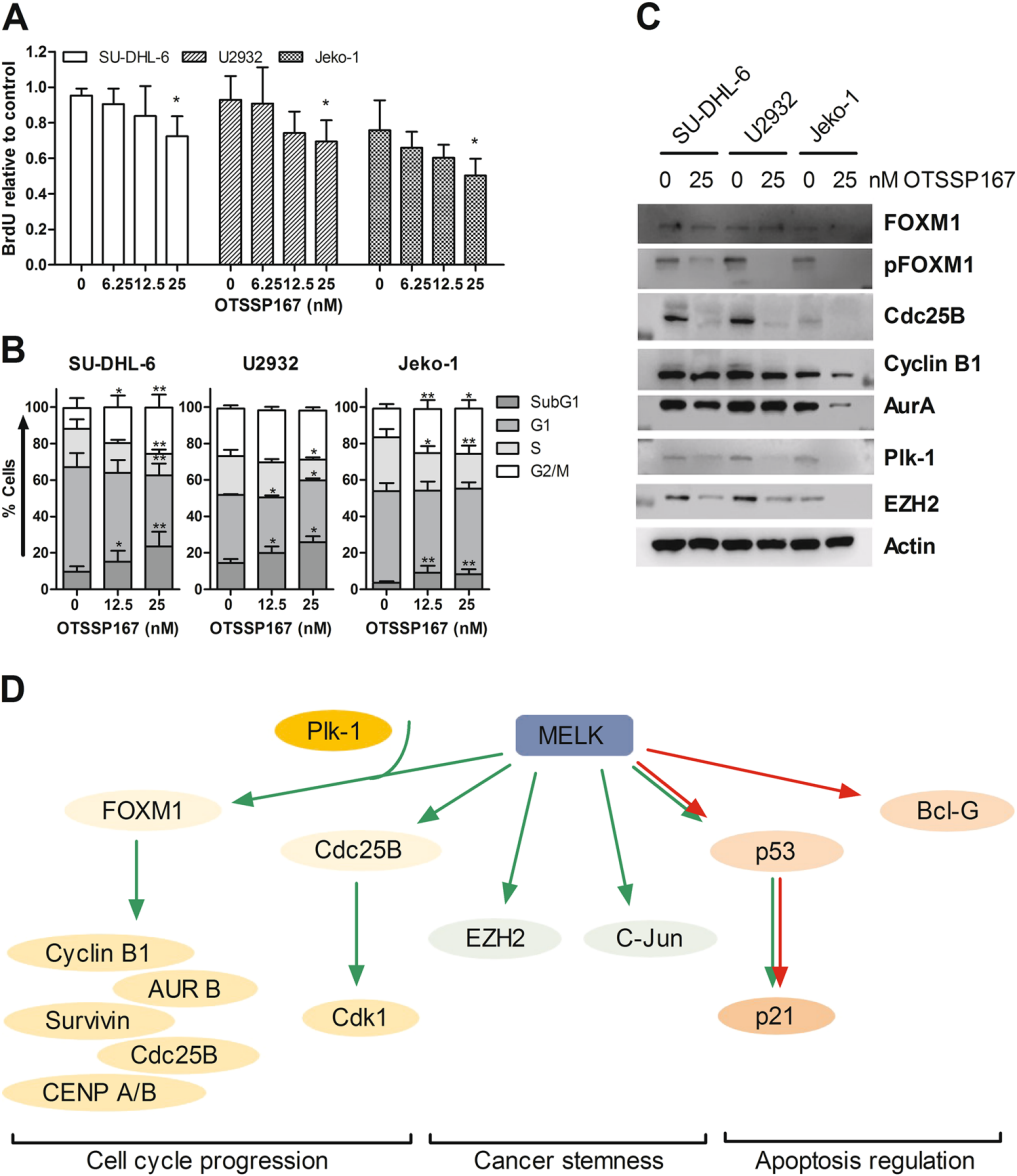
OTSSP167 treatment affects lymphoma cell cycle progression. **a** Proliferating lymphoma cells were incubated for 24 h with OTSSP167 (6.25, 12.5, and 25 nM) and subsequently with BrdU for 3 h and analyzed using the BrdU proliferation assay kit. Results shown are mean ± SD of three independent experiments. *p < 0.05. **b** Cell cycle analysis was performed after 24 h of OTSSP167 treatment (12.5 and 25 nM) using PI staining. Results shown are mean ± SD of three independent experiments. *p < 0.05 and **p < 0.01. **c** Protein levels of (p)FOXM1, Cdc25B, Cyclin B1, Aurora A kinase (AurA), Plk-1, and EZH2 were determined in DLBCL and MCL cell lines after 24 h of 25 nM OTSSP167 treatment. β-actin was used as loading control. One experiment representative of 3 is shown. **d** Scheme of the most examined downstream targets of MELK inhibition in cancer. Green arrows indicate activation and red arrows indicate inhibition/suppression. There is still some controversy whether MELK activates or inhibits p53 (AUR B: Aurora B kinase, CENP A/B: centromere protein A/B).

Since MELK has been shown to be involved in G2/M progression^8^, the effect of OTSSP167 on lymphoma cell proliferation was next evaluated using a BrdU incorporation assay. OTSSP167 treatment resulted in less proliferating DLBCL and MCL cells (Fig. 3a). Further examination of cell cycle progression revealed that OTSSP167 treatment resulted in a significant increase in the amount of cells in both subG1 and G2/M phase after 24 h (Fig. 3b). Together these data indicate that MELK also plays an important role in cell cycle progression in DLBCL and MCL and inhibition of MELK results in a prolonged G2/M phase.

To determine the underlying mechanism of action, the effect of OTSSP167 on the downstream targets (FOXM1, Cdc25B, and EZH2) was assessed by western blot. As shown in Fig. 3c, 24 h of OTSSP167 treatment strongly reduced the phosphorylation of FOXM1, while the total FOXM1 protein levels remained more or less stable. Moreover, Cdc25B and EZH2 protein levels were also strongly reduced in the OTSSP167-treated cells compared with the untreated samples. Since pFOXM1 is the activated transcription factor that regulates the expression of several mitotic regulators, the protein levels of cyclin B1, Aurora A kinase and Polo-like kinase 1 were also analyzed. Consistent with the reduced pFOXM1 levels, OTSSP167 treatment also decreased the protein levels of cyclin B1, Aurora A kinase, and Polo-like kinase 1 in all cell lines (Fig. 3c, d and Supplemental Fig. 2).

### OTSSP167 treatment reduces tumor growth in vivo and prolongs overall survival

**Fig. 4:**
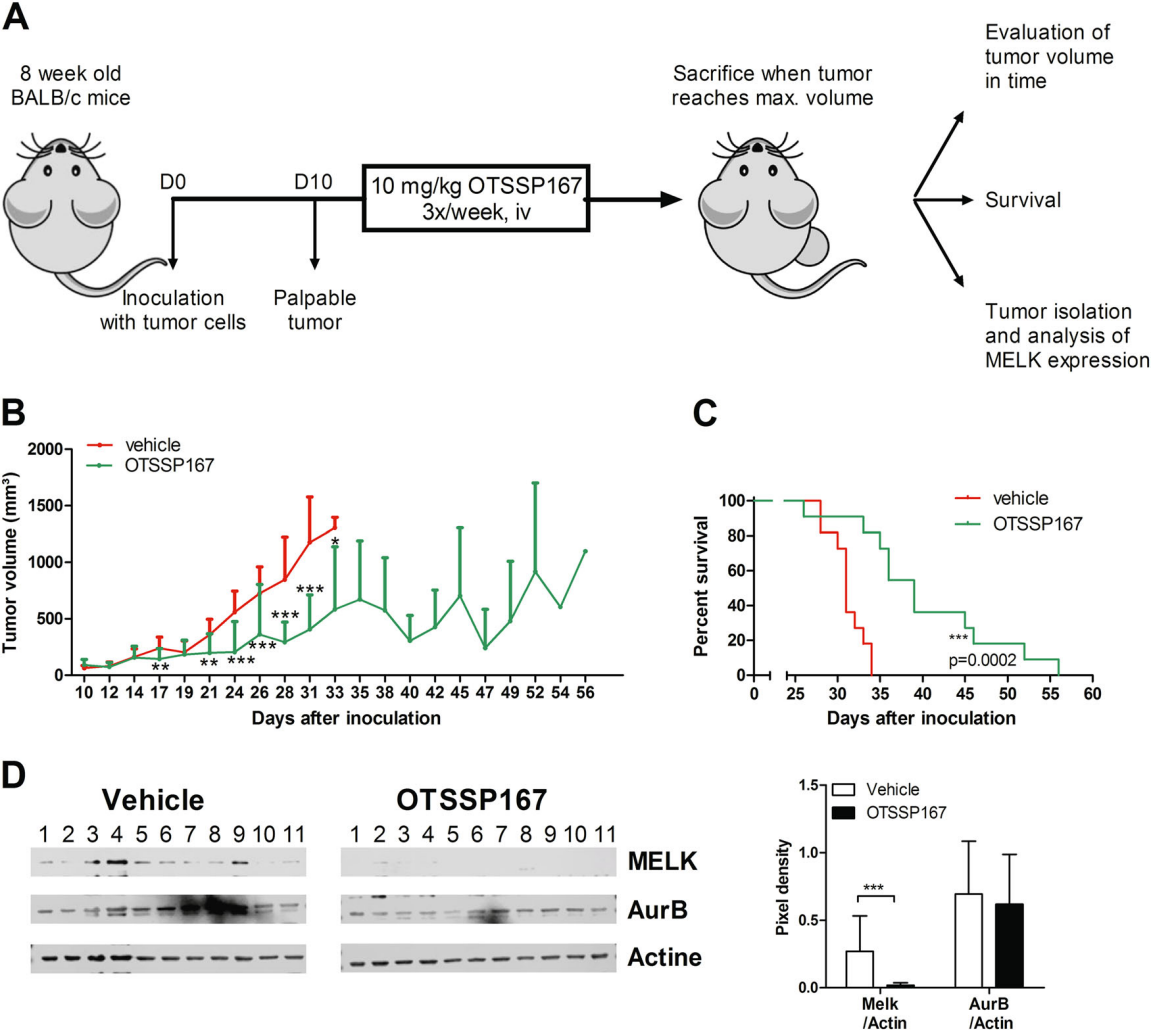
OTSSP167 treatment reduces tumor growth in vivo and prolongs overall survival. **a** Balb/c mice were inoculated with murine A20 lymphoma cells and when tumor was palpable the mice were treated with OTSSP167 (10 mg/kg). Mice were sacrificed when tumor volume reached 1500 mm³. **b** Evaluation of the tumor volume of vehicle (*n* = 11, red) and OTSSP167-treated mice (*n* = 11, green). *p < 0.05, **p < 0.01, and ***p < 0.001. **c** Survival curve of vehicle (red) and OTSSP167-treated mice (green). **d** Expression of MELK and Aurora B kinase (AurB) was determined in samples from vehicle and OTSSP167-treated mice. β-actin was used as loading control. Quantification was performed with Image J.

To validate the anti-lymphoma activity of OTSSP167 in vivo, the A20 syngeneic immunocompetent model of murine lymphoma was used. First, the sensitivity of the murine A20 lymphoma cells was evaluated in vitro. Consistent with the human cell lines, a dose-dependent decrease in viability and increase in apoptotic cells was observed after 48 h (Supplemental Fig. 3A–B). Next, 8-week-old Balb/c mice were subcutaneously inoculated with A20 lymphoma cells and when the tumor was palpable the OTSSP167 treatment started (Fig. 4a). The tumor volume (mm³) was assessed over time during this survival study. A significant difference in the tumor volume between vehicle-treated and OTSSP167-treated mice was already seen after 1 week of treatment (Fig. 4b). Moreover, all vehicle mice reached the maximum tumor volume within 34 days after inoculation, whereas OTSSP167-treated mice reached the maximum volume 56 days after inoculation (Fig. 4b and Supplemental Fig. 3C–D). This delay in tumor growth also correlated with a significant better survival for OTSSP167-treated mice compared with vehicle-treated mice (Fig. 4c). Western blot analysis confirmed that OTSSP167 treatment significantly reduced the MELK protein expression in samples obtained from the treated mice compared with vehicle mice (Fig. 4d), while no effect was seen on Aurora B kinase protein levels. Of note, the total body weight of the mice was not affected by the OTSSP167 treatment, suggesting that OTSSP167 is not toxic for the mice (Supplemental Fig. 3E).

### Combining OTSSP167 with venetoclax leads to a synergistic anti-lymphoma effect

**Fig. 5:**
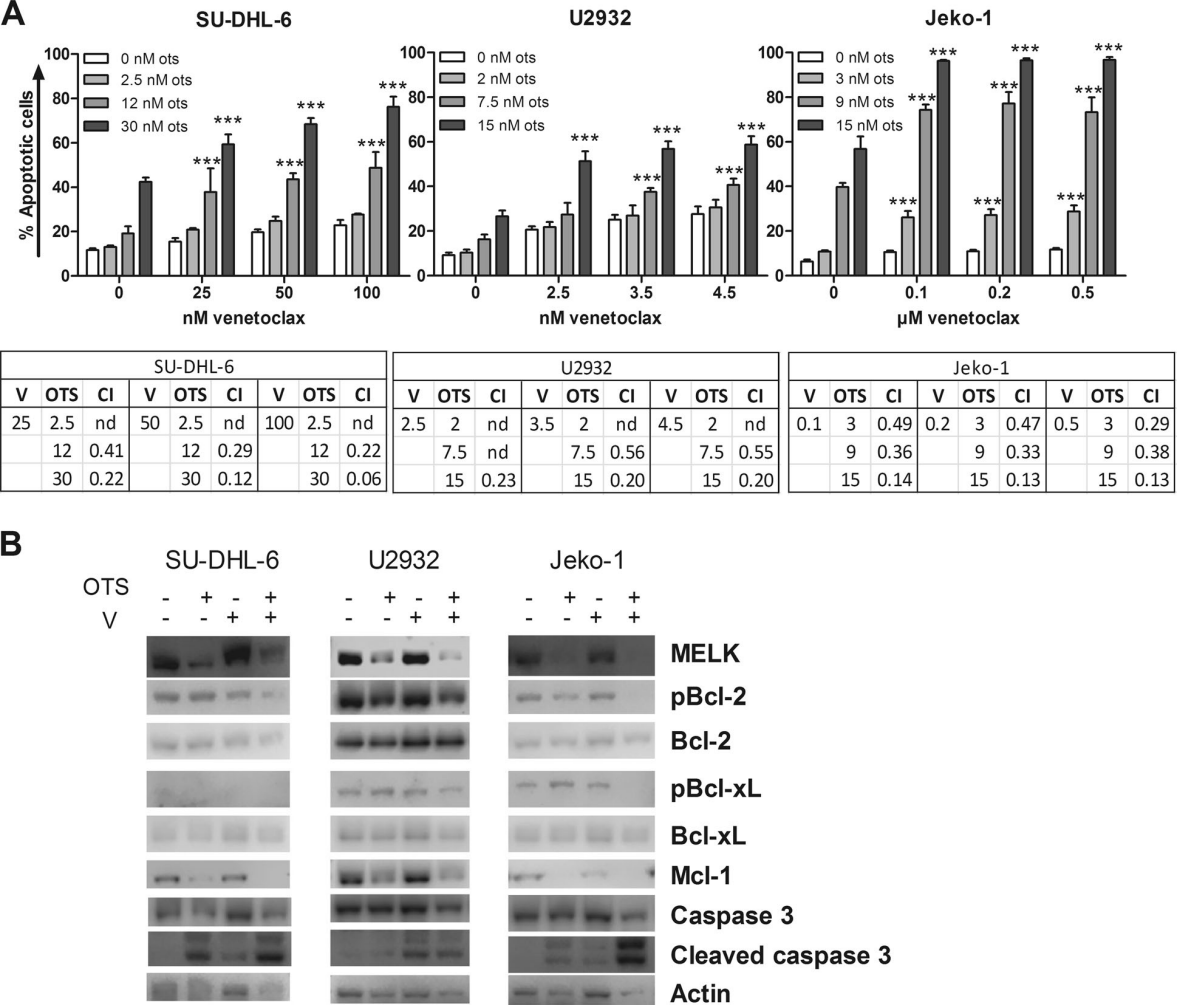
Combining OTSSP167 with venetoclax leads to a synergistic anti-lymphoma effect. **a** Apoptosis of OTSSP167 (ots) and venetoclax-treated SU-DHL-6, U2932, and Jeko-1 cells was determined after 48 h using an Annexin V/7’AAD staining followed by flow cytometry analysis. The sum of the percentage Annexin V and Annexin V/7’AAD positive cells are shown. Results shown are mean ± SD of four independent experiments. Each combination was compared with both single agents. ***p < 0.001 and combination indexes (CI) are shown. **b** Protein levels of MELK, (p)Bcl-2, (p)Bcl-xL, Mcl1, and caspase-3 were determined in DLBCL and MCL cell lines after 24 h of OTSSP167 treatment in combination with venetoclax. SU-DHL-6, U2932, and Jeko-1 cells were treated with, respectively, 30 nM OTSSP167 and 100 nM venetoclax, 15 nM OTSSP167 and 4.5 nM venetoclax, and 15 nM OTSSP167 and 500 nM venetoclax. β-actin was used as loading control. One experiment representative of 3 is shown.

Finally, OTSSP167 was combined with the standard-of-care agents doxorubicin and rituximab and the novel, selective Bcl-2 inhibitor venetoclax. Sub-optimal concentrations (IC-10, IC-30, and IC-50) of the agents were used to test their combinatory effect (Supplemental Table 2). OTSSP167 treatment did not significantly enhance the anti-lymphoma activity of doxorubicin nor rituximab in all lymphoma cell lines tested (Supplemental Fig. 4A–B). In contrast, OTSSP167 treatment was found to significantly and synergistically sensitize lymphoma cells of all subtypes tested to venetoclax-mediated cell death and this both at high and low concentrations (Fig. 5a). Next, the underlying mechanisms of action of the combination treatment was evaluated by western blot. As shown in Fig. 5b, a clear reduction in MELK and Mcl1 protein levels was observed in all DLBCL and MCL cell lines used after OTSSP167 treatment. No consistent differences were observed in (p)Bcl-2 and (p)Bcl-xL protein levels. Importantly, the combination of venetoclax and OTSSP167 also induced more apoptosis compared with both single agents, as evidenced by a further increase in the levels of cleaved caspase-3. Together, these data provide evidence that OTSSP167 treatment reduces Mcl1 protein levels, thereby sensitizing the lymphoma cells to venetoclax.

## Discussion

Despite the current improvements in the anti-lymphoma therapy, there is still a poor survival in one-third of the DLBCL patients and no difference is observed in overall survival of MCL patients^2,37^. Thus, it remains necessary to improve anti-lymphoma therapy. In this study, MELK is identified as a novel target in the aggressive lymphomas DLBCL and MCL. As described above, increased MELK expression was already reported in a variety of solid (breast, prostate, etc.) and hematological cancers (leukemia and multiple myeloma), and has been associated with a worse clinical outcome for these patients^6,14–18^. Consistent with these reports, MELK gene expression was also found significantly elevated in all DLBCL and MCL subtypes compared with their normal counterparts. Moreover, high MELK mRNA expression correlates with poor survival outcome in DLBCL patients. The data from our discovery cohort of 27 DLBCL patients suggest that high MELK protein expression could also be linked to a poor prognosis in DLBCL patients treated with R-CHOP. However, only a trend in differential MELK expression was observed in this small cohort; with only six patients having a poor prognosis according to the R-IPI scoring system. Further validation in a prospective study in larger patient cohorts thus remains warranted. Together, these data suggest that MELK could play a role in DLBCL and MCL pathogenesis, supporting the preclinical testing of MELK as novel target for DLBCL and MCL.

Targeting MELK in different cancer cell types, using either siRNA knockdown or pharmacological inhibition, resulted in an impaired tumor growth by inducing apoptosis and/or decreasing cell proliferation both in vitro and in vivo^11,15,17–24^. Several agents were already used to target MELK, such as MELK-T1^38^, MELK-8a^39^, MELK-IN-1^40^, and OTSSP167^41^. In our study OTSSP167 was used, since it is a potent MELK inhibitor that fits effectively into the active site of the kinase and is currently the only one under investigation in clinical trials^25,42^. Consistent with the previous studies in cancer, OTSSP167 reduces lymphoma cell viability in a dose-dependent manner and was able to induce caspase-3-mediated apoptosis. In addition, a dose-dependent decrease in cell proliferation is observed after OTSSP167 treatment, correlating with a prolonged G2/M phase in DLBCL and MCL. The anti-lymphoma activity of OTSSP167 was also validated in vivo using the murine A20 lymphoma model. A significant reduction in tumor growth was observed after OTSSP167 treatment, which was associated with a significant better overall survival of the OTSSP167-treated mice.

OTSSP167 has been described to inhibit MELK by blocking autophosphorylation of MELK, thus resulting in the degradation and loss of MELK protein^13^. It has also been described that OTSSP167 can have off-target effects on Aurora B kinase^8^. Accordingly, a clear decrease in MELK protein levels was observed in DLBCL and MCL cells after 24 h of OTSSP167 treatment. The protein levels of Aurora B kinase were also reduced, however, to a lesser extent. These findings were also confirmed in vivo, where MELK protein levels are again more affected by OTSSP167 treatment than Aurora B kinase protein levels. Concerning the underlying mechanisms, it was reported previously that both p53-dependent and p53-independent upregulation of p21 is an important part of the OTSSP167-induced apoptosis^7,12,13,43^. Moreover, it has recently been described that MELK interacts with the transcription factor FOXM1 (a master regulator for cell proliferation) in a Plk-1-dependent manner and that EZH2-mediated radiation resistance occurs through a MELK-FOXM1-dependent manner in glioblastoma cells^9,44^. FOXM1, Plk-1, and EZH2 are all described as interesting therapeutic targets in both DLBCL and MCL, and high expression of these genes is reported to be associated with poor survival in lymphoma patients^45–48^. Here it is shown that inhibiting MELK by OTSSP167 in DLBCL and MCL cells also causes a decrease in phosphorylation of FOXM1 and subsequent downregulation of the mitotic regulators Aurora A kinase, cyclin B1, Plk-1, and Cdc25B. The downregulation of these mitotic regulators can explain the observed prolonged G2/M phase after OTSSP167 treatment. In addition, EZH2 is also inhibited by the OTSSP167 treatment. In contrast to previous studies, no upregulation of p21 protein levels and no differences in p53 and phoshpo-p53 protein levels were observed (data not shown). However, loss of p53 appears to occur frequently in DLBCL patients^49^. The lymphoma cell lines used in this study are also characterized by either TP53 mutations or loss of expression of p53^50,51^, hence explaining the indifference in (phospho-) p53 protein levels after MELK inhibition. Consistent with our data, a study in p53-mutated myeloma cells also failed to observe an increase in p21 after OTSSP167 treatment^52^. Moreover, there is still some controversy whether the MELK-p53 interaction is pro- or anti-apoptotic in cancer cells. Several studies showed that MELK expression increases p53 activation, while others demonstrated an inverse correlation between MELK expression and p53 expression^7^. Since OTSSP167 is able to inhibit the expression and centrosome localization of Aurora B kinase, abrogation of the mitotic checkpoint (or spindle assembly checkpoint) could be an alternative mechanism of cell death by OTSSP167 treatment^8^. However, this remains to be confirmed in future studies.

To further improve the anti-lymphoma activity of OTSSP167, in vitro combination studies were performed. Previously, studies in multiple myeloma and breast cancer demonstrated that combining OTSSP167 with standard-of-care agents (including lenalidomide, pomalidomide, and bortezomib) could increase the anti-cancer effect of the standard-of-care agent^18,53^. A promising novel agent in clinical development in DLBCL and MCL is the selective Bcl-2 inhibitor venetoclax (also known as ABT-199). Bcl-2 seems to play a crucial role in the pathogenesis of these lymphomas justifying the use of venetoclax^54^. Although venetoclax demonstrated low single agent effects in clinical trials with B cell lymphomas, combination regimen (such as venetoclax and ibrutinib in refractory MCL or venetoclax and R-CHOP in DLBCL) suggest promising efficacy and good tolerability^54,55^. Mcl1 is considered a major determinant of resistance to venetoclax and Bolomsky et al. recently demonstrated that OTSSP167 treatment reduces the Mcl1 protein levels in MM cells^18,56^. Therefore, we hypothesized that OTSSP167 might sensitize the lymphoma cells to venetoclax by reducing Mcl1 levels. Consistent with this hypothesis, combining OTSSP167 with venetoclax significantly and highly synergistically enhances the anti-lymphoma effects of the MELK inhibitor in all cell lines tested, even in the highly venetoclax-sensitive ABC-DLBCL cell line U2932. Western blot analysis furthermore confirmed the reduction in Mcl1 protein levels in both OTSSP167 and combo treated cells and the further increase in cleaved caspase-3 levels in the combo treated cells. Importantly, it has been reported that a concentration of 1 µM venetoclax corresponds to the clinically recommended dose of 400 mg per day in patients^57,58^. For our in vitro combination experiments, we used sub-optimal concentrations of venetoclax well below 1 µM. This underlines the clinical relevance of our combination experiments. Since MELK inhibition in breast cancer was shown to activate the ATM-CHK2 pathway and thus suggested to reactivate the DNA damage response, we expected that combining OTSSP167 with doxorubicin would enhance the anti-lymphoma activity of this topoisomerase II inhibitor^38^. However, our data did not demonstrate any additional effect in DLBCL or MCL. A possible explanation could be the use of a different MELK inhibitor. In the former study MELK-T1 was used. This inhibitor was shown to induce a S phase arrest instead of the G2/M phase arrest observed here. Finally, OTSSP167 also failed to enhance the anti-lymphoma effect of the anti-CD20 monoclonal antibody rituximab. This might be explained by the minor cytotoxic effects that rituximab monotherapy had on the lymphoma cell lines in vitro.

In conclusion, our data show that high MELK expression is associated with poor prognosis in the aggressive B cell malignancy DLBCL. Targeting MELK using the small molecule OTSSP167 resulted in a downregulation of the mitotic regulators Cdc25B, cyclin B1, and Aurora kinases, resulting in a prolonged G2/M phase. Moreover, OTSSP167 demonstrated potent in vitro and in vivo anti-lymphoma activity, which was further enhanced by the Bcl-2 inhibitor venetoclax. These results therefore suggest MELK as a potential new target in the aggressive B cell lymphomas DLBCL and MCL, and support clinical testing of OTSSP167 in these cancers.

## Supplementary information

Supplemental information

Supplemental figure 1

Supplemental figure 2

Supplemental figure 3

Supplemental Figure 4

Supplemental table 1

Supplemental table 2
