# Supplementary material for: Maternal embryonic leucine zipper kinase is a novel target for diffuse large B cell lymphoma and mantle cell lymphoma
Source: Blood Cancer J. 2019 Nov 18;9(12):87. doi: 10.1038/s41408-019-0249-x (PMC6861269; doi:10.1038/s41408-019-0249-x)
Supplement: Supplementary file 1 — Supplemental information [file 41408_2019_249_MOESM1_ESM.docx]

**SUPPLEMENTAL INFORMATION**

**SUPPLEMENTAL FIGURES**

**Supplemental Figure 1: MELK expression in human lymphoma cell lines.**

(A) MELK gene expression levels of B cell samples (n=33) and different DLBCL (n=13) and MCL cell lines (n=5) was obtained from the GEP datasets GSE56315 and GSE36133. Mean expression ± SD is shown in red. *** p<0.001 (B-C) Basal MELK mRNA (B) and protein (C) levels were determined in isolated B cells, DLBCL (OCI-Ly1, OCI-Ly7, SU-DHL-6, RI-1 and U2932) and MCL cell lines (Mino, Jeko-1 and Rec-1) using quantitative real-time PCR and western blot respectively. Mean ± SD of 3 independent experiments is shown for quantitative real-time PCR and one experiment representative of 3 is shown for western blot. * p<0.05

**Supplemental Figure 2: Densitometry and normalization of protein levels to actin**

Protein levels of (p)FOXM1, Cdc25B, Cyclin B1, Aurora A kinase (AurA), Plk-1 and EZH2 were determined in DLBCL and MCL cell lines after 24 hours of 25nM OTSSP167 treatment. Quantification was performed with Image J.

**Supplemental Figure 3: OTSSP167 treatment on murine A20 lymphoma cells.**

(A-B) The murine A20 cell line was treated for 24 and 48 hours with OTSSP167 (3.125, 6.25, 12.5, 25 and 50 nM). The effect on viability was determined using a CellTiter-Glo assay (A). Results are shown as % viability relative to control. Results shown are mean ± SD of 3 independent experiments. The effect on apoptosis was determined using an Annexin V/7’-AAD staining followed by flow cytometric analysis (B). Percentage apoptotic cells are the sum of the percentage Annexin V and Annexin V/7’-AAD positive cells. Results shown are mean ± SD of 3 independent experiments. * p<0.05 (C-D) Evaluation of the tumor volume of each vehicle (C) and OTSSP167 treated mice (D). (E) Follow-up of the total body weight of vehicle (red) and OTSSP167 treated mice (green) in time.

**Supplemental Figure 4: OTSSP167 in combination with the standard of care agents doxorubicin and rituximab.**

(A-C) Apoptosis of OTSSP167 (ots) and doxorubicin (A), rituximab (B) or vincristine (C) treated SU-DHL-6, U2932 and Jeko-1 cells was determined after 48 hours using an Annexin V/7’AAD staining followed by flow cytometry analysis. The sum of the percentage Annexin V and Annexin V/7’AAD positive cells are shown. Results shown are mean ± SD of 4 independent experiments. Each combination was compared to both single agents. * p<0.05
