# Supplementary figures and images for: Maternal embryonic leucine zipper kinase is a novel target for diffuse large B cell lymphoma and mantle cell lymphoma
Source: Blood Cancer J. 2019 Nov 18;9(12):87. doi: 10.1038/s41408-019-0249-x (PMC6861269; doi:10.1038/s41408-019-0249-x)

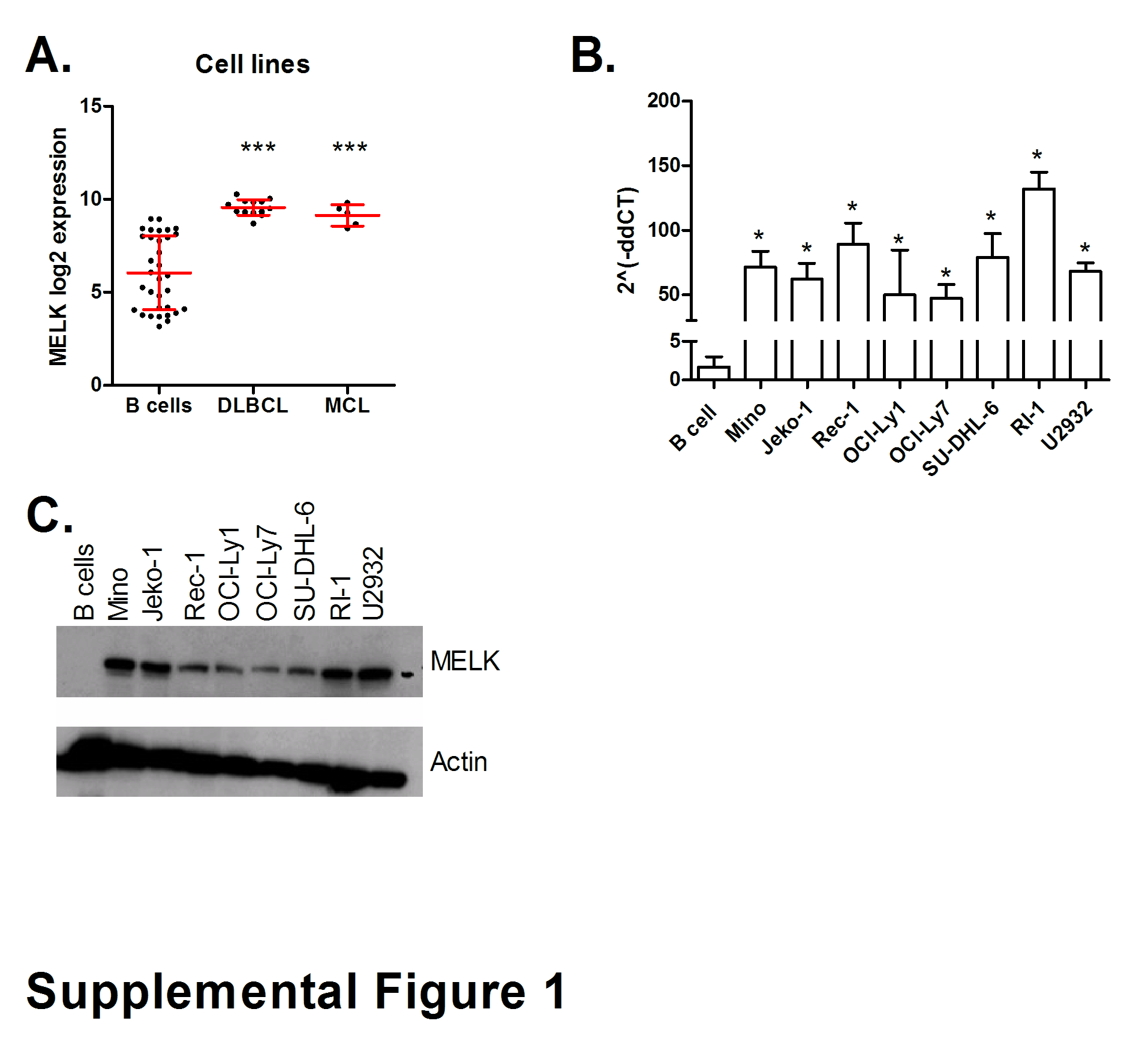

Supplement: Supplementary file 2 — Supplemental figure 1 [file 41408_2019_249_MOESM2_ESM.tif]

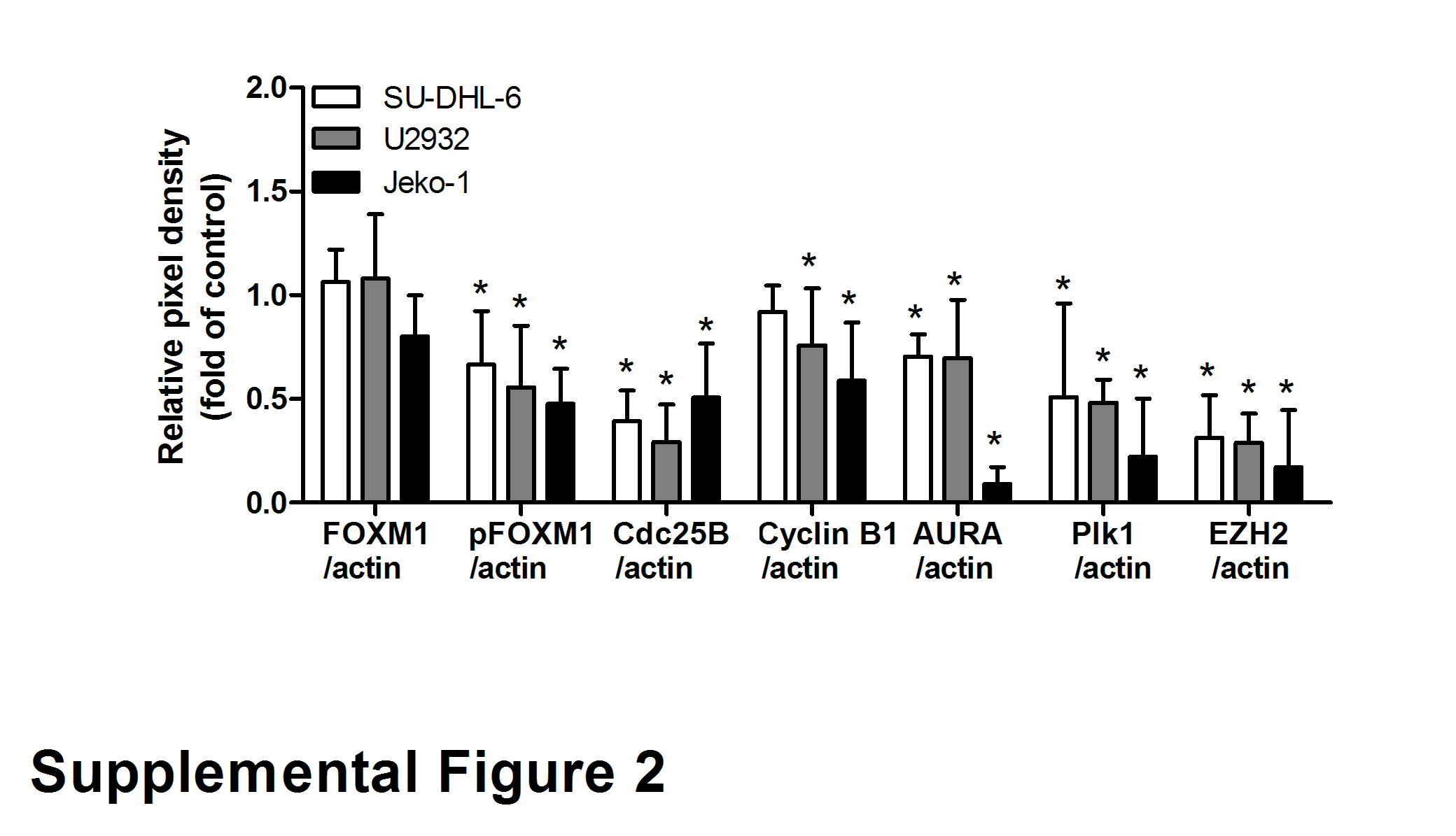

Supplement: Supplementary file 3 — Supplemental figure 2 [file 41408_2019_249_MOESM3_ESM.tif]

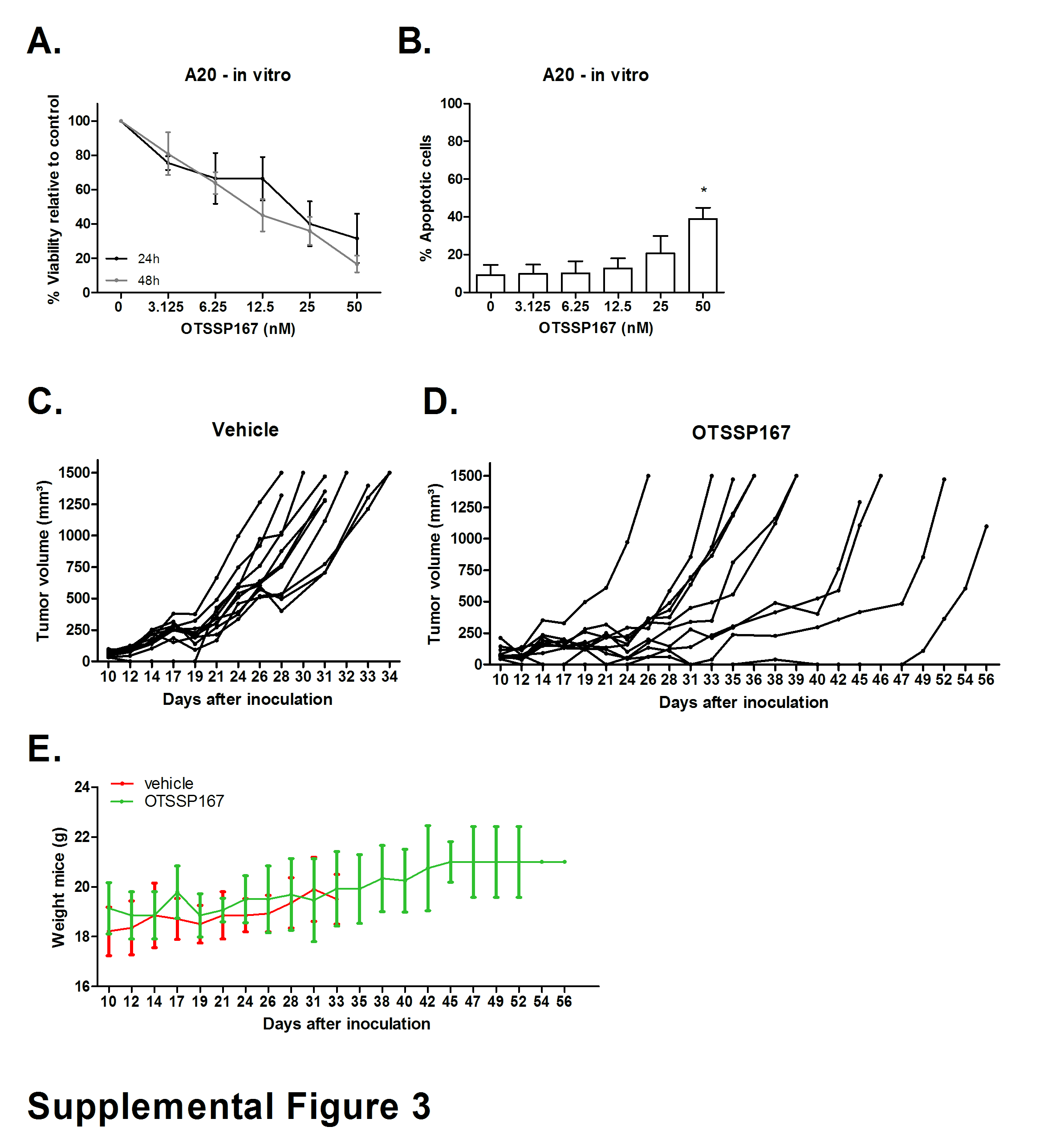

Supplement: Supplementary file 4 — Supplemental figure 3 [file 41408_2019_249_MOESM4_ESM.tif]

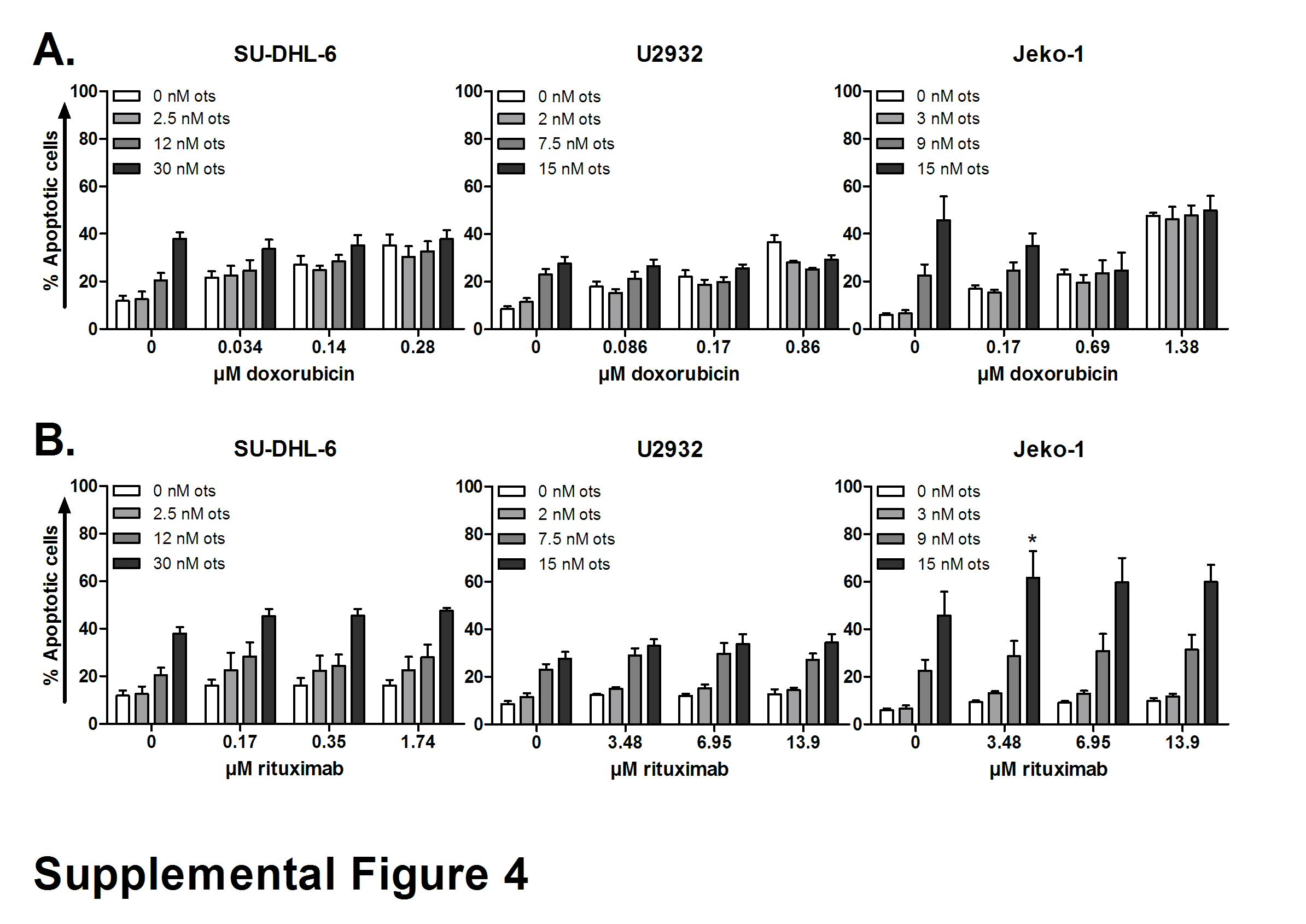

Supplement: Supplementary file 5 — Supplemental Figure 4 [file 41408_2019_249_MOESM5_ESM.tif]
