# Supplementary material for: Maternal embryonic leucine zipper kinase is a novel target for diffuse large B cell lymphoma and mantle cell lymphoma
Source: Blood Cancer J. 2019 Nov 18;9(12):87. doi: 10.1038/s41408-019-0249-x (PMC6861269; doi:10.1038/s41408-019-0249-x)
Supplement: Supplementary file 6 — Supplemental table 1 [file 41408_2019_249_MOESM6_ESM.docx]

**SUPPLEMENTAL TABLE 1**

| **A.** |  | **IC-50 (nM)** |
| --- | --- | --- |
|  | Mino | 13.445 |
|  | Jeko-1 | 15.293 |
|  | Rec-1 | 25.330 |
|  | OCI-Ly1 | 13.133 |
|  | OCI-Ly7 | 18.165 |
|  | SU-DHL-6 | 30.383 |
|  | RI-1 | 6.414 |
|  | U2932 | 15.410 |

| **B.** |  | **Mino** | **Jeko-1** | **RI-1** | **U2932** | **SU-DHL-6** | **Rec-1** | **OCI-Ly1** | **OCI-Ly7** |
| --- | --- | --- | --- | --- | --- | --- | --- | --- | --- |
|  | MELK |  |  |  |  |  |  |  |  |
|  | IC-10 | 2.5 | 3 | 1.2 | 2 | 2.5 | 2.5 | 2.5 | 8 |
|  | IC-30 | 7.5 | 9 | 3.5 | 7.5 | 12 | 12 | 7.5 | 14 |
|  | IC-50 | 13 | 15 | 6 | 15 | 30 | 25 | 13 | 18 |

| High protein expression |  |
| --- | --- |
| Low protein expression |  |

| **C.** |  | **Jeko-1** | **OCI-Ly1** | **OCI-Ly7** | **Mino** | **U2932** | **SU-DHL-6** | **RI-1** | **Rec-1** |
| --- | --- | --- | --- | --- | --- | --- | --- | --- | --- |
|  | Doubling time | 28±8 | 28.5±0.8 | 28.5±0.8 | 32.5±4.9 | 30±8.5 | 41±1.5 | 35.5±10 | 36±1.5 |
|  | IC-10 | 3 | 2.5 | 8 | 2.5 | 2 | 2.5 | 1.2 | 2.5 |
|  | IC-30 | 9 | 7.5 | 14 | 7.5 | 7.5 | 12 | 3.5 | 12 |
|  | IC-50 | 15 | 13 | 18 | 13 | 15 | 30 | 6 | 25 |

| High doubling time |  |
| --- | --- |
| Intermediate doubling time |  |
| Low doubling time |  |

**Supplemental Table 1:** The IC-50 values for OTSSP167 for the different cell lines after 48 hours of treatment (A). MELK protein expression (B) and doubling time (hours/generation, C) was measured and compared to the sensitivity of OTSSP-167 (IC-10, IC-30 and IC-50 (nM)) in each cell line.
