# Supplementary material for: Maternal embryonic leucine zipper kinase is a novel target for diffuse large B cell lymphoma and mantle cell lymphoma
Source: Blood Cancer J. 2019 Nov 18;9(12):87. doi: 10.1038/s41408-019-0249-x (PMC6861269; doi:10.1038/s41408-019-0249-x)
Supplement: Supplementary file 7 — Supplemental table 2 [file 41408_2019_249_MOESM7_ESM.docx]

**SUPPLEMENTAL TABLE 2**

|  | **SU-DHL-6** | | | **U2932** | | | **Jeko-1** | | |
| --- | --- | --- | --- | --- | --- | --- | --- | --- | --- |
| Reagent | IC-10 | IC-30 | IC-50 | IC-10 | IC-30 | IC-50 | IC-10 | IC-30 | IC-50 |
| OTSSP167 (nM) | 2.5 | 12 | 30 | 2 | 7.5 | 15 | 3 | 9 | 15 |
| Doxorubicin (µM) | 0.034 | 0.14 | 0.28 | 0.086 | 0.17 | 0.86 | 0.17 | 0.69 | 1.38 |
| Rituximab (µM) | 0.17 | 0.35 | 1.74 | 3.48 | 6.95 | 13.9 | 3.48 | 6.95 | 13.9 |
| Venetoclax (µM) | 0.025 | 0.05 | 0.1 | 0.0025 | 0.0035 | 0.0045 | 0.1 | 0.2 | 0.5 |

**Supplemental Table 2:** The IC-10, IC-30 and IC-50 values for OTSSP167, doxorubicin, rituximab and venetoclax for the different cell lines after 48 hours of treatment, measured with cell viability assay.
